# Three Month Follow-Up of Patients With COVID-19 Pneumonia Complicated by Pulmonary Embolism

**DOI:** 10.3389/fmolb.2021.809186

**Published:** 2022-02-03

**Authors:** Cecilia Calabrese, Anna Annunziata, Martina Flora, Domenica Francesca Mariniello, Valentino Allocca, Maria Ilaria Palma, Antonietta Coppola, Ilernando Meoli, Pia Clara Pafundi, Giuseppe Fiorentino

**Affiliations:** ^1^ Department of Translational Medical Sciences, University of Campania “Luigi Vanvitelli”, Naples, Italy; ^2^ Department of Intensive Care, A.O.R.N dei Colli, Naples, Italy; ^3^ GEMELLI GENERATOR - Facility of Epidemiology and Biostatistics, Fondazione Policlinico Gemelli, Roma, Italy

**Keywords:** COVID - 19, pulmonary embolism, lung function, spirometry, DLCO, KCO, six minute walk test

## Abstract

**Background:** Previous studies have demonstrated persistent dyspnoea and impairment of respiratory function in the follow-up of patients who have recovered from COVID-19 pneumonia. However, no studies have evaluated the clinical and functional consequences of COVID-19 pneumonia complicated by pulmonary embolism.

**Objective:** The aim of our study was to assess the pulmonary function and exercise capacity in COVID-19 patients 3 months after recovery from pneumonia, either complicated or not by pulmonary embolism.

**Methods:** This was a retrospective, single-centre, observational study involving 68 adult COVID-19 patients with a positive/negative clinical history of pulmonary embolism (PE) as a complication of COVID-19 pneumonia. Three months after recovery all patients underwent spirometry, diffusion capacity of the lungs for carbon monoxide (DLCO), and 6 minute walk test (6MWT). In addition, high-resolution computed tomography (HRCT) of the lung was carried out and CT-pulmonary angiography was conducted only in the PE+ subgroup. Patients with a previous diagnosis of PE or chronic lung diseases were excluded from the study.

**Results:** Of the 68 patients included in the study, 24 had previous PE (PE+) and 44 did not (PE−). In comparison with the PE− subgroup, PE+ patients displayed a FVC% predicted significantly lower (87.71 ± 15.40 vs 98.7 ± 16.7, *p* = 0.009) and a significantly lower DLCO% predicted (*p* = 0.023). In addition, a higher percentage of patients were dyspnoeic on exercise, as documented by a mMRC score ≥1 (75% vs 54.3%, *p* < 0.001) and displayed a SpO_2_ <90% during 6MWT (37.5% vs 0%, *p* < 0.001). HRCT features suggestive of COVID-19 pneumonia resolution phase were present in both PE+ and PE− subjects without any significant difference (*p* = 0.24) and abnormalities at CT pulmonary angiography were detected in 57% of the PE+ subgroup.

**Conclusion:** At the 3 month follow-up, the patients who recovered from COVID-19 pneumonia complicated by PE showed more dyspnoea and higher impairment of pulmonary function tests compared with those without PE.

## Introduction

Coronavirus disease 2019 (COVID-19), caused by the novel Severe Acute Respiratory Syndrome CoronaVirus-2 (SARS-CoV-2), can involve multiple organs, though the lungs’ involvement plays the key role in all the most severe clinical manifestations. After SARS-CoV-2 accesses human cells by the angiotensin-converting enzyme 2 (ACE2) receptors, mostly expressed by type 2 pneumocytes, an acute systemic inflammatory response may occur, followed by several lung pathological events (Mo et al., 2020). The extensive injury of alveolar epithelial cells and endothelial cells can elicit a fibroproliferative response. Chronic alveolar and vascular remodeling can also, in turn, evolve either in lung fibrosis and/or pulmonary hypertension (Frija-Masson et al., 2020; Venkataraman and Frieman, 2017). In addition, the interplay between inflammation and thrombosis, known as thrombo-inflammation, may contribute to a procoagulant state, which can be responsible for the vascular thrombosis frequently detected in small caliber pulmonary vessels (Bikdeli et al., 2020; Goeijenbier et al., 2012; Klok et al., 2020). Venous thromboembolism has been reported particularly in severe COVID 19 patients, with a prevalence ranging from 17 to 69%, and genetic risk factors seem to play a pathogenetic role (Calabrese et al., 2021). While the clinical manifestations of patients affected by COVID-19 during the acute phase of the disease have been largely described, the consequences after recovery from SARS-Cov-2 infection still need to be further investigated.

The assessment of lung injury in COVID-19 survivors includes different types of functional respiratory evaluations, among which the most commonly used are spirometry, diffusing capacity of the lung for carbon monoxide (DLCO), and 6-minute walk test (6MWT) (British Thoracic Society Guidance, 2020). In accordance with the most recent guidelines of the British Thoracic Society, a face-to-face review is suggested 12 weeks after discharge in patients with severe COVID-19 pneumonia. Several studies, performed either at discharge or several months after recovery, have shown that the most common respiratory functional abnormality is reduced DLCO, followed by a restrictive ventilatory defect at the spirometry (Mo et al., 2020; Torres-Castro et al., 2021). In addition, an impairment of exercise capacity has also been described in a small percentage of patients post COVID-19 (Vitacca et al., 2021). However, no study has focused yet on the respiratory functional consequences occurring in patients surviving COVID-19 pneumonia complicated by pulmonary embolism, except for Mendez et al. (2021) who observed only fifteen patients with worse DLCO values.

The present study aimed to assess pulmonary function and exercise capacity in COVID-19 patients 3 months after recovery from pneumonia, either complicated or not, by pulmonary embolism.

## Materials and Methods

We conducted a retrospective, single-centre, observational study involving adult patients who received a diagnosis of SARS-CoV-2 pneumonia confirmed by real time reverse transcription polymerase chain reaction (RT-PCR) on naso-pharyngeal swab and high resolution computed tomography (HRCT) of the lung. All enrolled patients had either severe COVID-19 pneumonia (in the presence of fever, cough, dyspnea, fast breathing, one among respiratory rate >30 breaths/min, severe respiratory distress, or SpO_2_ <90% on room air) or critical with mild ARDS (P/F between 200 and 300 mmHg, with either PEEP or cPAP ≥5 cm H_2_O) (SARI Guidelines, 2019)*.*

Patients included in the study were divided into two subgroups based on the positive/negative clinical history of pulmonary embolism (PE) as a complication of COVID-19 pneumonia, i.e. PE+ and PE−, respectively. PE diagnosis was confirmed by CT pulmonary angiography. All patients were treated with the best of care according to the NIH COVID 19 guidelines (NIH Covid-19 Guidelines, 2019), including prophylactic dose anticoagulation unless contraindicated. Patients with COVID-19 who experienced an incident thromboembolic event were managed with therapeutic doses of anticoagulant therapy. After discharge, PE+ patients were treated with direct oral anticoagulants (DOACs) for at least 6 months. Following hospital discharge till follow-up visit, all patients did not need any clinical visits or hospital admission. Three months after recovery from SARS-CoV-2 infection, all patients performed pulmonary function tests at the outpatient service of the Department of Respiratory Pathophysiology, Monaldi Hospital, Naples (Italy), including spirometry, DLCO, and 6MWT. In addition, HRCT of the lung was carried out and CT pulmonary angiography was performed only in the PE + subgroup.

Based on the clinical data collected from medical records about all clinical comorbidities and ongoing therapies, confirmed by lung HRCT, we excluded from the study all patients with a previous diagnosis of chronic lung diseases (i.e., chronic obstructive pulmonary disease, bronchial asthma, diffuse parenchymal lung disease) or PE.

All subjects provided written informed consent to participate in the study, which was approved by the local ethics committee of the University of Campania Luigi Vanvitelli and A.O.R.N. Ospedali dei Colli, in accordance with the 1976 Declaration of Helsinki and its later amendments (AOC-0020053-2020).

### Data Collection

Baseline demographic and anthropometric characteristics (sex, age and body mass index), history of smoking, and comorbidities were collected from clinical medical records.

Spirometry and single-breath DLCO test were performed according to the American Thoracic Society/European Respiratory Society (ATS/ERS) guidelines (Graham et al., 2017, 2019), using Vyntus BODY (Vyaire Medical).

The following spirometric parameters were measured: forced expiratory volume in the first second (FEV_1_), forced vital capacity (FVC), FEV_1_/FVC ratio and forced expiratory flow at 25, 50, 75% of the forced vital capacity (FEF 25, FEF 50 and FEF 75, respectively). DLCO, alveolar volume (V_A_), and transfer coefficient of the lung for carbon monoxide (KCO) were measured by the single-breath DLCO test.

All parameters were expressed as absolute values and percentages of the predicted value (%)and considered reduced if below the lower limit of normality (LLN), according to the Global Lung Function Initiative 2012 reference equations for spirometry (Quanjer et al., 2012).

An obstructive ventilatory defect was defined by a FEV_1_/FVC ratio lower than LLN. A flow-volume curve displaying an FVC % <80% and an FEV_1_/FVC ≥70% was considered suggestive of a restrictive ventilatory defect (Soriano et al., 2012). Reduced DLCO % and KCO % were defined as lower than 80% of the predicted value, according to the Global Lung Function Initiative 2017 reference equations for DLCO (Stanojevic et al., 2017).

Proper performance of spirometry and single-breath DLCO test was ensured by medical personnel and all the measures suggested by local national guidelines to avoid the risk of SARS-CoV2 infection were adopted (Tognella and Piccioni, 2021). In particular, the measurement of lung volumes by plethysmography, due to the objective difficulties in the disinfection of the chamber in the post-pandemic phase 2, was not allowed. All enrolled patients had to exhibit a negative nasopharyngeal swab for the molecular detection of SARS-CoV-2 RNA before pulmonary functional exams.

The 6 minute walk test (6MWT) was performed according to ATS/ERS guidelines (Holland et al., 2014). The distance walked during 6 minutes was measured and compared with 6MWT predictive values according to the reference equation published by ENRIGHT and SHERRILL (Enright and Sherrill, 1998) and considered reduced if lower than LLN. The dyspnea intensity was assessed by the modified Medical Research Council (mMRC) dyspnea scale (range from 0- dyspnea only with strenuous exercise to 4- too dyspneic to leave the house or breathless when dressing) (Fletcher et al., 1959; Williams, 2017). Finally, the Borg dyspnea scale score (range from 0 – nothing at all to 10 very severe) was assessed before and after 6MWT. During the test, peripheral capillary oxygen saturation (SpO_2_) was measured by pulse oximetry on the index finger and SpO_2_ levels below 90% were considered pathological.

### Statistical Analysis

All variables included in the study were first analyzed by descriptive statistics techniques. In depth, qualitative data were expressed as number and percentage, while quantitative variables either median and interquartile range (IQR) or mean and standard deviation (SD), based on their distribution, were assessed by the Shapiro-Wilk test. Between the groups, the differences at baseline were tested either by the parametric paired Student *t* Test or by the non-parametric Wilcoxon signed rank test, as appropriate, whilst qualitative data were analyzed either by the Chi Square test or the Fisher Exact test. The most significant findings were further exemplified by box-plots and bar diagrams.

A *p*-value <0.05 was considered statistically significant. Data were analyzed using SPSS Software, Version 26 (IBM, Armonk, New York, United States), and STATA 16 software (StataCorp. 2019: StataCorp LP, College Station, TX, United States).

## Results

In total, 68 COVID-19 patients were enrolled in the study, 24 with PE (PE+) and 44 without (PE−). All baseline clinical and functional characteristics of the whole study population are described in Table 1. The mean age was 54.9 (±12.8) years and the majority of patients were males (73.5%), nonsmokers (61.8%), and overweight (median BMI 28). The most frequent comorbidities were systemic arterial hypertension (45.6%) and obesity (41.2%) and 39 (57.4%) patients showed more than one comorbidity. The median time from the recovery from SARS-CoV-2 infection to the clinical follow-up visit was 90 days (IQR 60–120).

**TABLE 1:** Baseline characteristics of the study population (*n* = 68).

Age (yrs) (mean ± SD)	54.9 (12.8)
Sex (M/F), *n* (%)	50 (73.5)/18 (26.5)
BMI, median [IQR]	28 [25–31]
Smoking habit, *n* (%)	
Yes	2 (2.9)
No	42 (61.8)
Ex	24 (35.3)
Comorbidities, *n* (%)^a^	
Arterial hypertension	31 (45.6)
Cardiomyopathy	5 (7.4)
Diabetes Mellitus	10 (14.7)
Obesity	28 (41.2)
GERD	15 (22.1)
Spirometry, *n* (%)	
FEV_1_% (mean ± SD)	94.5 (17.9)
FVC % (mean ± SD)	94.8 (17)
FEV_1_/FVC (mean ± SD)	81.8 (6.7)
FEF 25% (mean ± SD)	100.2 (26)
FEF 50%, median [IQR]	95.5 [76–110]
FEF 75%, median [IQR]	73.5 [53.5–89.5]
Interpretation of Spirometry, *n* (%)	
Normal	58 (85.3)
Restrictive deficit	6 (8.8)
Obstructive deficit	4 (5.9)
DiffusionCapacity Test	
DLCO %, median [IQR]	82 [72.3–93]
DLCO % < 80, *n* (%)	27 (39.7)
KCO % (mean ± SD)	99.9 (19.2)
KCO% < 80%, *n* (%)	8 (11.8)
DLCO % < 80% + KCO <80%, *n* (%)	7 (10.3)
DLCO % < 80% + KCO >80%, *n* (%)	20 (29.4)
6MWT	
mMRC ≥1, *n* (%)	37 (54.4)
SpO_2_ <90% 6MWT, *n* (%)	9 (13.2)
Walk distance<LLN, *n* (%)	17 (25)
Lung HRCT, *n* (%)	
Normal	12 (17.6)
Pathologic	45 (66.3)
Not performed	11 (6.1)
Interval discharge and respiratory function test (days), median (IQR)	90 (60–120)

SD, standard deviation; M, Male; F, Female; BMI, Body Mass Index; IQR, Interquartile range; GERD, Gastroesophageal Reflux Disease; FEV1, Forced expiratory volume in one second; FVC, Forced Vital Capacity; FEF 25, FEF 50 and FEF 75, forced expiratory flow at 25, 50, 75% of the forced vital capacity; DLCO, diffusion capacity of the lungs for carbon monoxide; KCO, transfer coefficient of the lung for carbon monoxide; mMRC, modified Medical Research Council Dyspnea scale; 6MWT, 6-minute walk test; SpO_2_, peripheral capillary oxygen saturation; LLN, lower limit of normality; HRCT, high resolution computed tomography.

a Comorbidities prevalence was computed considering singularly each comorbidity.

Spirometry and DLCO were uneventfully completed in all subjects. Abnormalities at spirometry were detected in a small percentage of patients (14.7%), with 4 (5.9%) showing an obstructive ventilatory defect and 6 (8.8%) displaying a flow-volume curve suggestive of a restrictive ventilatory defect. A reduction of DLCO <80% predicted was instead observed in 27 (39.7%) patients and 7 (10.3%) showed a concomitant reduction of KCO <80%, while 20 (29.4%) had a normal KCO.

The 6MWT was performed in all study population. Most patients (37, 54.4%) complained of dyspnea, assessed by an mMRC grade ≥1. Moreover, 13.2% of patients displayed a SpO_2_ <90% during the test, and 25% had a distance walked below the LLN. All patients, except for three, complained of increased dyspnea after the test, as rated by the Borg dyspnea scale.

Altogether, 57 of the 68 patients performed HRCT of the lung. Radiological changes suggestive of a resolution phase of COVID-19 pneumonia, represented by residual areas of ground glass opacity (GGO) and/or consolidations and/or linear bands, were present in 78.9% of patients.

The comparison of PE+ and PE− subgroups did not disclose any significant difference for sex and comorbidities. PE+ patients, indeed, were significantly older than PE− patients (61 ± 11.1 vs 51.5 ± 12.5, *p* = 0.003) and also had a higher prevalence of smoking habits (*p* = 0.015) (Table 2). In addition, we did not observe any significant difference between PE+ and PE− subgroups in the prevalence of patients with severe or critical with mild ARDS COVID-19 pneumonia (8 vs 15% and 92 vs 85%, *p* = 0.476, respectively).

**TABLE 2:** Comparison of baseline characteristics between PE+ and PE− subgroups.

	PE+ (*n* = 24)	PE− (*n* = 44)	*p*
Age (yrs) (mean ± SD)	61 (11.1)	51.5 (12.5)	0.003
Sex (M/F), *n* (%)	19(79.2)/5(20.8)	31(70.5)/13(29.5)	0.436
BMI, median [IQR]	28.5 [26–32.5]	27.5 [25–31]	0.624
Smoking habit, *n* (%)			0.015
Yes	2 (8.3)	—	
No	10 (41.7)	32 (72.7)	
Ex	12 (50)	12 (27.3)	
Comorbidities, *n* (%)			
Arterial hypertension	14 (58.3)	17 (38.6)	0.119
Cardiomyopathy	3 (12.5)	2 (4.5)	0.475
Diabetes Mellitus	6 (25)	4 (9.1)	0.158
Obesity	10 (41.7)	18 (40.9)	0.952
GERD	4 (16.7)	11 (25)	0.627
Spirometry, *n* (%)			
FEV_1_% (mean ± SD)	91.1 (15.5)	97.9 (18.9)	0.140
FVC % (mean ± SD)	87.7 (15.4)	98.7 (16.7)	0.009
FVC % <80% (%)	29.1	6.8	0.033
FEV_1_/FVC (mean ± SD)	83.6 (7.9)	80.9 (5.8)	0.117
FEF 25% (mean ± SD)	100.2 (25.3)	100.3 (26.7)	0.989
FEF 50%, median [IQR]	99.5 [79.5–108.5]	89.5 [69.3–112]	0.458
FEF 75%, median [IQR]	76.5 [59–82.3]	72 [52.5–94]	0.940
Interpretation of spirometry, *n* (%)			0.091
Normal	20 (83.3)	38 (86.4)	
Restrictive deficit	4 (16.7)	2 (4.5)	
Obstructive deficit	—	4 (9.1)	
Diffusion capacity test			
DLCO %, median [IQR]	79.5 [61–89.5]	86.5 [75.3–95.5]	0.023
DLCO % <80, *n* (%)	12 (50)	15 (34.1)	0.200
KCO % (mean ± SD)	95.2 (18.2)	102.3 (19.4)	0.160
KCO% < 80%, *n* (%)	4 (16.7)	4 (9.1)	0.354
DLCO% <80% + KCO<80%, *n* (%)	4 (16.7)	3 (6.8)	0.390
DLCO% <80% + KCO>80%, *n* (%)	8 (33.3)	12 (27.3)	0.600
6MWT			
mMRC ≥1, *n* (%)	18 (75)	19 (54.3)	<0.001
SpO_2_ <90% 6MWT, *n* (%)	9 (37.5)	0	<0.001
Walking distance<LLN, *n* (%)	3 (16.7)	14 (40)	0.088
Lung HRCT, *n* (%)^a^			0.244
Normal	2 (10)	10 (27)	
Pathologic	18 (90)	27 (73)	

SD, standard deviation; M, Male; F, Female; BMI, Body Mass Index; IQR, interquartile range; GERD, Gastroesophageal Reflux Disease; FEV1, Forced expiratory volume in one second; FVC, Forced Vital Capacity; FEF 25, FEF 50 and FEF 75, forced expiratory flow at 25, 50, 75% of the forced vital capacity; DLCO, diffusion capacity of the lungs for carbon monoxide; KCO, transfer coefficient of the lung for carbon monoxide; mMRC, modified Medical Research Council Dyspnea scale; 6MWT, 6-minute walk test; LLN, lower limit of normality; HRCT, high resolution computed tomography; SpO_2_, peripheral capillary oxygen saturation.

a We considered only the real number of patients (57) who performed the exam.

**FIGURE 1:**
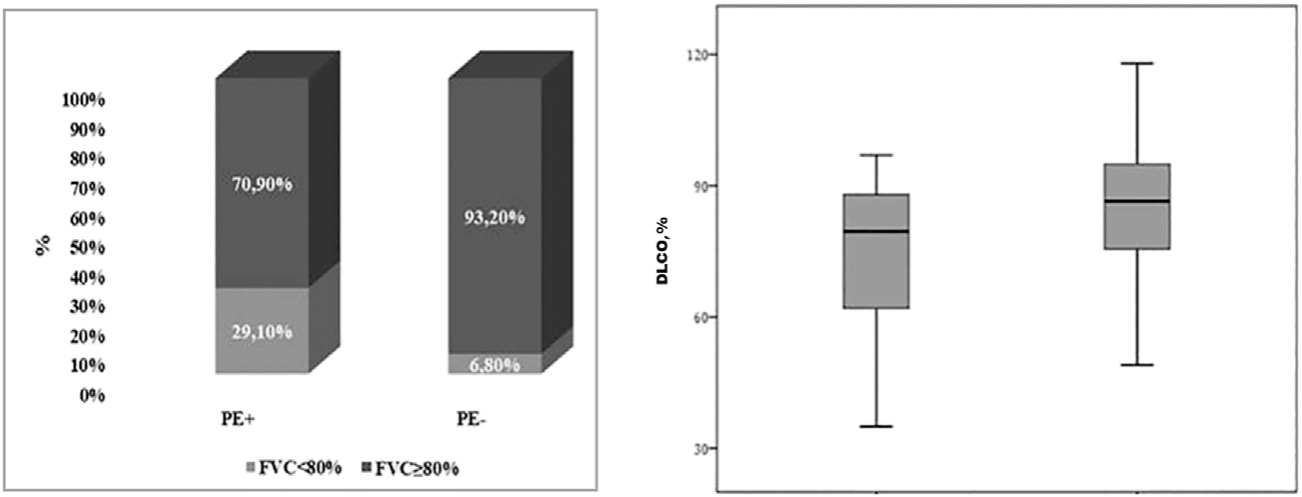
Box-plot showing differences between PE+ AND PE− in terms of FVC% <80% (29.1 vs 6.8%, *p* = 0.033) (on the left) and in terms of DLCO% [79.5 (61–89.5) vs 86.5 (75.3–95.5); *p* = 0.023] (on the right). PE, Pulmonary Embolism; FVC, Forced Vital Capacity; DLCO, Diffusion capacity of the lungs for carbon monoxide; LLN, Lower Limit of Normality.

The results of the pulmonary functional tests in the two subgroups of patients are described in Table 2. At the spirometry, PE+ patients displayed a FVC% predicted significantly lower than PE− (87.71 ± 15.40 vs 98.7 ± 16.7, *p* = 0.009). In particular, a higher percentage of PE+ patients had a FVC% <80% in comparison with PE− (29.1 vs 6.8%, *p* = 0.033) (Figure 1, left panel). Moreover, the flow volume curve suggestive of a restrictive ventilator defect was more prevalent in PE + compared to PE− subgroup, although without reaching a statistical significance (16.7% vs 4.5%, *p* = 0.212). PE+ patients also showed a significantly lower DLCO % predicted (*p* = 0.023) (Figure 1, right panel). Instead, no statistical difference in KCO% was observed between PE+ and PE−, though a higher percentage of PE+ patients had a concomitant reduction of DLCO% and KCO%.

**FIGURE 2:**
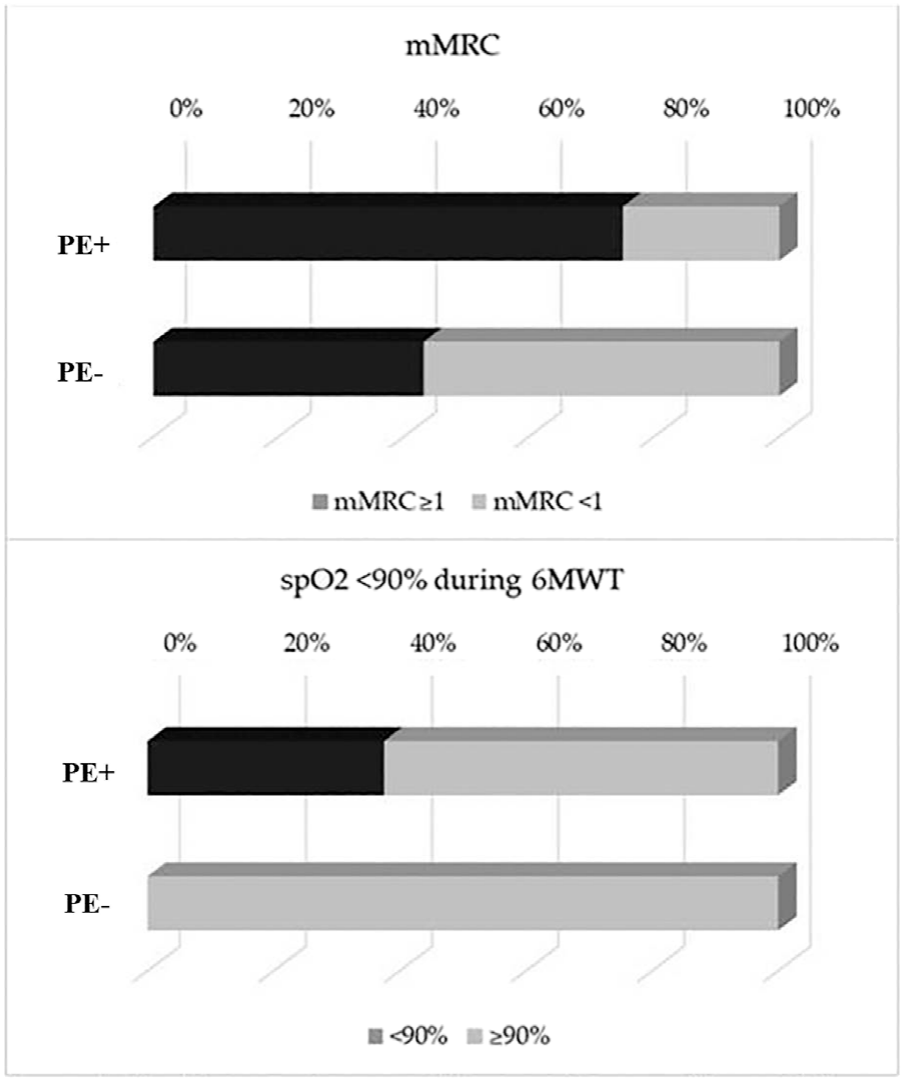
Bar diagram showing differences between PE+ AND PE− in terms of mMRC ≥1 (75 vs 54.3%, *p* < 0.001) (upper panel) and for SpO_2_ <90% during 6-minute walk test (6MWT) (37.5 vs 0%, *p* < 0.001) (lower panel). PE, Pulmonary Embolism; mMRC, modified Medical Research Council Dyspnea scale; 6MWT, 6-minute walk test.

The main symptom complained by all COVID-19 survivors was persistent dyspnea, which was significantly more prevalent in PE+ as compared to PE− subgroup, as demonstrated by the mMRC dyspnea score ≥1 (75% vs 54.3%, *p* < 0.001) (Figure 2, upper panel).

We also observed a higher prevalence of PE+ patients with a SpO_2_<90% during 6MWT (37.5 vs 0%, *p* < 0.001) (Figure 2, lower panel). The percentage of patients with a distance walked at 6MWT< LLN, indeed, did not significantly differ between the two subgroups. Finally, HRCT features suggestive of COVID-19 pneumonia resolution phase were present in 90% of PE+ patients and 73% of PE− subjects (*p* = 0.24), whilst abnormalities at CT pulmonary angiography were detected in 57% of PE+.

## Discussion

The main objective of the present study was to assess potential differences in pulmonary functional impairment in COVID-19 survivors with pneumonia either complicated or not by PE. At the 3 month follow-up, COVID-19 survivors with previous PE showed a significantly lower FVC% and DLCO%. Although a higher percentage of PE+ patients had a concomitant reduction of DLCO% and KCO%, the difference between the two subgroups did not reach statistical significance. Recently, several authors have discussed the clinical significance of the reduction of DLCO and whether it is associated with a decrease in KCO among COVID-19 survivors.

DLCO represents the lung’s ability to exchange gas and may occur with various combinations of KCO and VA, each suggesting different underlying pathological modifications. KCO, the transfer coefficient of the lung for carbon monoxide, is a measure of CO uptake from alveolar gas and is affected by the thickness and area of the alveolar-capillary membrane, blood volume, and hemoglobin concentration/properties in capillary vessels supplying ventilated alveoli. A reduced DLCO with either normal or near normal KCO might be related to a reduced alveolar volume caused by changes in the mechanical properties of the chest wall and respiratory muscles. Conversely, when both DLCO and KCO are reduced, we might suspect either membrane or pulmonary capillary abnormalities (Nusair, 2020). Unexpectedly, in the PE+ subgroup, we did not find any significantly higher prevalence of patients showing a reduction of both DLCO % and KCO %, although abnormalities suggestive of pulmonary embolism at CT pulmonary angiography were present after 3 months in this subset of patients. This finding might be due to the relatively small sample size of our study population, with only 10% of them showing functional abnormalities. In addition, Laveneziana et al. (2021) have suggested that reduction of both DLCO and KCO could be in favor of lesions involving alveolar membrane and/or pulmonary capillaries susceptible of some recovery. On the contrary, the reduction of the only DLCO with normal KCO suggests definitive alveolar loss destruction with no perspective of recovery. Of note, in the majority of both PE+ and PE− subjects the two functional parameters were within the normal ranges, whilst about one-third displayed a reduction of only DLCO with normal KCO. Similarly, Mo X and colleagues, who evaluated at the discharge 110 COVID-19 survivors classified into three groups of severity, observed a higher reduction of both DLCO and total lung capacity in the most severe cases. In about half of patients with reduced DLCO, KCO remained within the normal ranges (Mo et al., 2020).

The most frequently observed ventilatory deficit, though in a small percentage of patients, was the restrictive, with a higher percentage of PE+ patients displaying a reduced forced vital capacity. However, a restrictive ventilatory deficit might be associated with the condition of obesity, which was frequently observed in COVID-19 patients and documented in the present study in more than 40% of both PE+ and PE− patients. Moreover, it is of note that the majority of COVID-19 pneumonia survivors did not have any clinically significant changes in spirometry at 3 month follow-up.

Regarding the exercise test, indeed, we observed a significantly higher prevalence of PE+ patients complaining of dyspnea on exertion and peripheral oxygen desaturation during the test. Similarly, another study showed, in COVID-19 survivors with persistent dyspnea, a lower % predicted walked distance and oxygen saturation during the 6MWT, alongside higher ratings of dyspnea and leg fatigue during the exercise test (Cortés-Telles et al., 2021). According to the inclusion criteria, all patients enrolled in the study did not have any previous respiratory diseases, which could have affected their pulmonary function and/or exercise capacity, even though smoking history was more prevalent in PE+ patients, also older than PE−. In addition, no findings suggestive of concomitant lung diseases were observed at lung HRCT both in PE+ and PE− patients. All COVID-19 survivors showed residual imaging abnormalities of an ongoing resolution of COVID-19 pneumonia at lung HRCT, though without differences between PE+ and PE− subgroups. These data suggest that the higher clinical and functional impairment of PE+ patients could be a consequence of a pulmonary perfusion defect persisting 3 months after the recovery rather than to pulmonary parenchymal alterations. Findings from CT pulmonary angiography demonstrated perfusion defect in 57% of PE + patients.

Frija-Masson et al. (2020) found abnormal lung function tests in more than 50% of patients with a mix of restrictive ventilatory defects and low diffusion patterns. In about one third of patients, the authors observed that a decreased DLCO was not associated with chest CT abnormalities, thus leading to the hypothesis of vascular damage induced by SARS-CoV-2. Huang et al. (2021) further observed no significant correlation between total severity score at chest CT and pulmonary functional parameters during follow-up visits. The authors also observed a reduction of DLCO in more than 50% of patients, with a higher incidence of DLCO impairment associated with a lower percentage of predicted TLC and 6MWD in severe as compared to moderate and mild disease. The authors further observed that a small percentage of patients with no residual abnormalities presented with a slight decrease in DLCO.

Our study is of course characterized by several limitations. First, the relatively small sample size and the short follow-up mean that the results need to be interpreted carefully. As previously stated, the cost of the nasopharyngeal swab for the molecular detection of SARS-CoV-2 RNA, which was needed to perform the pulmonary function tests, limited the number of patients who agreed to the study participation. As for follow-up, we performed further PFTs 18 months after the recovery from SARS-Cov2 infection in 10 PE+ and PE− patients of the 27 who displayed a reduction of FVC% and/or DLCO at 3 month follow-up. Overall, seven patients saw an improvement of DLCO, which reached normal percentage predicted values in four cases, while the impairment remained stable in three patients. One patient also had a reduction of FVC% predicted value, which returned at normal values at 18-month follow-up (data not published). Based on these findings, we can hypothesize that most patients should experience recovery from functional impairment. Finally, the diagnosis of restrictive pattern exclusively performed on the reduced FVC associated with either a normal or higher FEV_1_/FVC is questionable. However, the measurement of TLC by plethysmography was avoided due to the restrictions imposed by local national guidelines regarding pulmonary function tests during the COVID-19 pandemic (Tognella and Piccioni, 2021).

These limitations suggest the need for larger study populations and longer follow-up studies to better establish the characteristics and trends of modification of lung function and exercise tolerance in COVID-19 survivors complicated by PE. However, based on our findings, which depict a higher pulmonary functional impairment at 3-month follow-up among patients with a history of pulmonary embolism complicating SARS-CoV2 pneumonia, we suggest using diagnostic exams to assess the presence of pulmonary embolism in COVID-19 survivors with persistent dyspnea, DLCO impairment, and peripheral oxygen desaturation during the 6MWT.

## Data Availability

The raw data supporting the conclusions of this article will be made available by the authors, without undue reservation.

## References

[B1] Bikdeli B. Madhavan M. v. Jimenez D. Chuich T. Dreyfus I. Driggin E. (2020). COVID-19 and Thrombotic or Thromboembolic Disease: Implications for Prevention, Antithrombotic Therapy, and Follow-Up. J. Am. Coll. Cardiol. 75 (23), 2950–2973. 10.1016/j.jacc.2020.04.031 32311448 PMC7164881

[B2] British Thoracic Society Guidance (2020). British Thoracic Society Guidance on Respiratory Follow up of Patients with a Clinico-Radiological Diagnosis of COVID-19 Pneumonia. Available at: https://www.brit-thoracic.org.uk/document-library/quality-improvement/covid-19/resp-follow-up-guidance-post-covid-pneumonia/ .

[B3] Cortés-Telles A. López-Romero S. Figueroa-Hurtado E. Pou-Aguilar Y. N. Wong A. W. Milne K. M. (2021). Pulmonary Function and Functional Capacity in COVID-19 Survivors with Persistent Dyspnoea. Respir. Physiol. Neurobiol. 288, 103644. 10.1016/j.resp.2021.103644 33647535 PMC7910142

[B4] Enright P. L. Sherrill D. L. (1998). Reference Equations for the Six-Minute Walk in Healthy Adults. Am. J. Respir. Crit. Care Med. 158 (5), 1384–1387. 10.1164/ajrccm.158.5.9710086 9817683

[B5] Fletcher C. M. Elmes P. C. Fairbairn A. S. Wood C. H. (1959). Significance of Respiratory Symptoms and the Diagnosis of Chronic Bronchitis in a Working Population. BMJ 2 (5147), 257–266. 10.1136/bmj.2.5147.257 13823475 PMC1990153

[B6] Frija-Masson J. Debray M. P. Gilbert M. Lescure F. X. Travert F. Borie R. (2020). Functional Characteristics of Patients with SARS-CoV-2 Pneumonia at 30 days post-infection. Eur. Respir. J. 56 (2), 2001754. 10.1183/13993003.01754-2020 32554533 PMC7301832

[B7] Goeijenbier M. van Wissen M. van de Weg C. Jong E. Gerdes V. E. A. Meijers J. C. M. (2012). Review: Viral Infections and Mechanisms of Thrombosis and Bleeding. J. Med. Virol. 84 (10), 1680–1696. 10.1002/jmv.23354 22930518 PMC7166625

[B8] Graham B. L. Brusasco V. Burgos F. Cooper B. G. Jensen R. Kendrick A. (2017). 2017 ERS/ATS Standards for Single-Breath Carbon Monoxide Uptake in the Lung. Eur. Respir. J. 49 (1), 1600016. 10.1183/13993003.00016-2016 28049168

[B9] Graham B. L. Steenbruggen I. Miller M. R. Barjaktarevic I. Z. Cooper B. G. Hall G. L. (2019). Standardization of Spirometry 2019 Update. An Official American Thoracic Society and European Respiratory Society Technical Statement. Am. J. Respir. Crit. Care Med. 200 (8), e70–e88. 10.1164/rccm.201908-1590ST 31613151 PMC6794117

[B10] Holland A. E. Spruit M. A. Troosters T. Puhan M. A. Pepin V. Saey D. (2014). An Official European Respiratory Society/American Thoracic Society Technical Standard: Field Walking Tests in Chronic Respiratory Disease. Eur. Respir. J. 44 (6), 1428–1446. 10.1183/09031936.00150314 25359355

[B11] Huang C. Huang L. Wang Y. Li X. Ren L. Gu X. (2021). 6-Month Consequences of COVID-19 in Patients Discharged from Hospital: A Cohort Study. Lancet 397, 10270. 10.1016/S0140-6736(20)32656-8 PMC7833295 33428867

[B12] Klok F. A. Kruip M. J. H. A. van der Meer N. J. M. Arbous M. S. Gommers D. A. M. P. J. Kant K. M. (2020). Incidence of Thrombotic Complications in Critically Ill ICU Patients with COVID-19. Thromb. Res. 191, 145–147. 10.1016/j.thromres.2020.04.013 32291094 PMC7146714

[B13] Laveneziana P. Sesé L. Gille T. (2021). Pathophysiology of Pulmonary Function Anomalies in COVID-19 Survivors. Breathe 17 (3), 210065. 10.1183/20734735.0065-2021 35035546 PMC8753644

[B14] Mendez R. Latorre A. González-Jiménez P. Feced L. Bouzas L. Yépez K. (2021). Reduced Diffusion Capacity in COVID-19 Survivors. Ann. Am. Thorac. Soc. 18, 1253. 10.1513/AnnalsATS.202011-1452RL 33472019 PMC8328367

[B15] Mo X. Jian W. Su Z. Chen M. Peng H. Peng P. (2020). Abnormal Pulmonary Function in COVID-19 Patients at Time of Hospital Discharge. Eur. Respir. J. 55 (6), 2001217. 10.1183/13993003.01217-2020 32381497 PMC7236826

[B16] NIH Covid-19 Guidelines (2019). COVID-19 Treatment Guidelines Panel. Coronavirus Disease 2019 (COVID-19) Treatment Guidelines. National Institutes of Health. Available at: https://www.covid19treatmentguidelines.nih.gov/ (April 21, 2020). 34003615

[B17] Nusair S. (2020). Abnormal Carbon Monoxide Diffusion Capacity in COVID-19 Patients at Time of Hospital Discharge. Eur. Respir. J. 56 (1), 2001832. 10.1183/13993003.01832-2020 32703822 PMC7376285

[B18] Quanjer P. H. Stanojevic S. Cole T. J. Baur X. Hall G. L. Culver B. H. (2012). Multi-ethnic Reference Values for Spirometry for the 3-95-yr Age Range: The Global Lung Function 2012 Equations. Eur. Respir. J. 40 (6), 1324–1343. 10.1183/09031936.00080312 22743675 PMC3786581

[B19] SARI Guidelines (2019). Clinical Management of Severe Acute Respiratory Infection (SARI) when COVID-19 Disease is Suspected: Interim Guidance, 13 March 2020. Available at: https://apps.who.int/iris/handle/10665/331446 .

[B20] Soriano J. B. Miravitlles M. García-Río F. Muñoz L. Sánchez G. Sobradillo V. (2012). Spirometrically-defined Restrictive Ventilatory Defect: Population Variability and Individual Determinants. Prim. Care Respir. J. 21 (2), 187–193. 10.4104/pcrj.2012.00027 22430039 PMC6547920

[B21] Stanojevic S. Graham B. L. Cooper B. G. Thompson B. R. Carter K. W. Francis R. W. (2017). Official ERS Technical Standards: Global Lung Function Initiative Reference Values for the Carbon Monoxide Transfer Factor for Caucasians. Eur. Respir. J. 50 (3), 1700010. 10.1183/13993003.00010-2017 28893868

[B22] Tognella S. Piccioni P. (2021). Le prove di funzionalità respiratoria nell’era della pandemia da COVID-19. Position Paper AIPO – ITS. Available at: http://www.aiponet.it/news/speciale-covid-19/2471-le-prove-di-funzionalita-respiratoria-nell-era-della-pandemia-da-covid-19-position-paper-aipo-its.html .

[B23] Torres-Castro R. Vasconcello-Castillo L. Alsina-Restoy X. Solis-Navarro L. Burgos F. Puppo H. (2020). Respiratory Function in Patients post-infection by COVID-19: A Systematic Review and Meta-Analysis. Pulmonology 27 (4), 328. 10.1016/j.pulmoe.2020.10.013 33262076 PMC7687368

[B24] Venkataraman T. Frieman M. B. (2017). The Role of Epidermal Growth Factor Receptor (EGFR) Signaling in SARS Coronavirus-Induced Pulmonary Fibrosis. Antiviral Res. 143, 142–150. 10.1016/j.antiviral.2017.03.022 28390872 PMC5507769

[B25] Vitacca M. Paneroni M. Brunetti G. Carlucci A. Balbi B. Spanevello A. (2021). Characteristics of COVID-19 Pneumonia Survivors with Resting Normoxemia and Exercise-Induced Desaturation. Respir. Care 66 (11), 1657–1664. 10.4187/respcare.09029 34429351

[B26] Williams N. (2017). The MRC Breathlessness Scale. Occup. Med. 67 (6), 496–497. 10.1093/occmed/kqx086 28898975

